# Oleoylethanolamide exerts anti-inflammatory effects on LPS-induced THP-1 cells by enhancing PPARα signaling and inhibiting the NF-κB and ERK1/2/AP-1/STAT3 pathways

**DOI:** 10.1038/srep34611

**Published:** 2016-10-10

**Authors:** Lichao Yang, Han Guo, Ying Li, Xianglan Meng, Lu Yan, Dan Zhang, Sangang Wu, Hao Zhou, Lu Peng, Qiang Xie, Xin Jin

**Affiliations:** 1 Xiamen Key Laboratory of Chiral Drugs, Medical College, Xiamen University, Xiamen, Fujian, 361102, P. R. China; 2 Department of Pharmacology, Xiamen Medical College, Xiamen, Fujian, 361008, P. R. China; 3 Department of Cardiology, the First Affiliated Hospital of Xiamen University, Xiamen, Fujian, 361003, P. R. China; 4 Xiamen Cancer Center, Department of Radiation Oncology, the First Affiliated Hospital of Xiamen University, Xiamen, Fujian, 361003, P. R. China

## Abstract

The present study aimed to examine the anti-inflammatory actions of oleoylethanolamide (OEA) in lipopolysaccharide (LPS)-induced THP-1 cells. The cells were stimulated with LPS (1 μg/ml) in the presence or absence of OEA (10, 20 and 40 μM). The pro-inflammatory cytokines were evaluated by qRT-PCR and ELISA. The THP-1 cells were transiently transfected with PPARα small-interfering RNA, and TLR4 activity was determined with a blocking test using anti-TLR4 antibody. Additionally, a special inhibitor was used to analyse the intracellular signaling pathway. OEA exerted a potent anti-inflammatory effect by reducing the production of pro-inflammatory cytokines and TLR4 expression, and by enhancing PPARα expression. The modulatory effects of OEA on LPS-induced inflammation depended on PPARα and TLR4. Importantly, OEA inhibited LPS-induced NF-κB activation, IκBα degradation, expression of AP-1, and the phosphorylation of ERK1/2 and STAT3. In summary, our results demonstrated that OEA exerts anti-inflammatory effects by enhancing PPARα signaling, inhibiting the TLR4-mediated NF-κB signaling pathway, and interfering with the ERK1/2-dependent signaling cascade (TLR4/ERK1/2/AP-1/STAT3), which suggests that OEA may be a therapeutic agent for inflammatory diseases.

Inflammation is closely related to the development of vascular diseases, such as restenosis, hypertension, and atherosclerosis. Lipopolysaccharide (LPS) is the major constituent of the outer wall of gram-negative bacteria and plays a critical role in mediating inflammation. Toll-like receptor 4 (TLR4) is a pattern-recognizing receptor that recognizes exogenous ligands such as bacterial LPS. It is well established that LPS triggers inflammatory and immune responses mainly through the TLR4 receptor. The activation of TLR4 by LPS induces the activation of nuclear factor-κB (NF-κB) and mitogen-activated protein kinase (MAPK) pathways and ultimately results in the release of pro-inflammatory cytokines^1^. NF-κB activation promotes the rapid phosphorylation of IκBs by the IKK signalosome complex^2^, which subsequently results in the translocation of the free NF-κB subunit to the nucleus where it binds to the promoter regions of target genes and induces the transcription of pro-inflammatory mediators including tumour necrosis factor-alpha (TNF-α), interleukin-1β (IL-1β) and IL-6. MAPK pathways include intracellular signaling molecules that are involved in the TLR4 signal transduction pathway which is responsible for the production of inflammatory mediators^3^. Signal transducer and activator of transcription-3 (STAT3) has been demonstrated to play a vital role in the inflammatory signaling cascade triggered by LPS. Monocytes/macrophages are involved in mediating inflammation. When monocytes/macrophages are stimulated with LPS, TLR4 expression is markedly increased, which triggers pro-inflammatory cytokine production^4^. These pro-inflammatory cytokines play important roles in inflammatory diseases. THP-1 is a human monocytic cell line derived from an acute monocytic leukaemia patient. THP-1 cells are notably sensitive to LPS and respond by expressing many inflammatory cytokines. In the present study, LPS-stimulated THP-1 cells were used as a model of inflammation *in vitro*.

Peroxisomal proliferator-activated receptor alpha (PPARα) is a ligand-activated transcription factor that is widely expressed in various cell types and tissues^5^. PPARα plays a major role in regulating inflammatory processes and has emerged as a popular drug target for hyperlipidaemia and inflammation in recent years^6^,^7^,^8^. Activation of PPARα by its agonists can inhibit LPS-induced increases in pro-inflammatory mediators, such as TNF-α, IL-1β, Cox-2, and IL-6, in a variety of cell types^9^,^10^. Additionally, the PPARα activator fenofibrate inhibits inflammatory responses through the TLR4-dependent signaling pathway and decreases serum levels of pro-inflammatory mediators in atherosclerotic patients^11^. Therefore, modulation of the TLR4-dependent signaling pathway may be a new therapeutic strategy for the treatment of inflammatory diseases.

Oleoylethanolamide (OEA) is produced by the small intestine. The biosynthesis of OEA is modulated by bile acids^12^. A previous study demonstrated that OEA is a potent endogenous ligand of PPARα^13^. The administration of OEA, as a pharmacological drug, modulates feeding, glucose homeostasis, lipid metabolism, neuroprotection and anti-atherosclerotic functions via the activation of the PPARα signaling pathway^13^,^14^,^15^,^16^,^17^. Furthermore, our latest study demonstrated that OEA treatment improves spatial cognitive deficits by enhancing hippocampal neurogenesis after transient focal cerebral ischemia^18^. Fenofibrate, a synthetic PPARα agonist with a range of anti-inflammatory properties, decreases serum levels of pro-inflammatory cytokines in atherosclerotic patients^19^,^20^ and exerts anti-inflammatory properties by antagonizing LPS-induced inflammatory responses in vascular smooth muscle cells^21^. Therefore, fenofibrate was used as a positive control in the present study. Although OEA is an endogenous ligand of PPARα that which mediates anti-inflammatory effects via the inhibition of inflammatory signaling pathways, little is known about the inhibitory effects of OEA on LPS-induced inflammatory responses or the underlying mechanisms involved. The aim of this study was to examine the anti-inflammatory actions and mechanisms of OEA in LPS-induced THP-1 cells.

## Results

### Effects of OEA on cell viability

**Figure 1:**
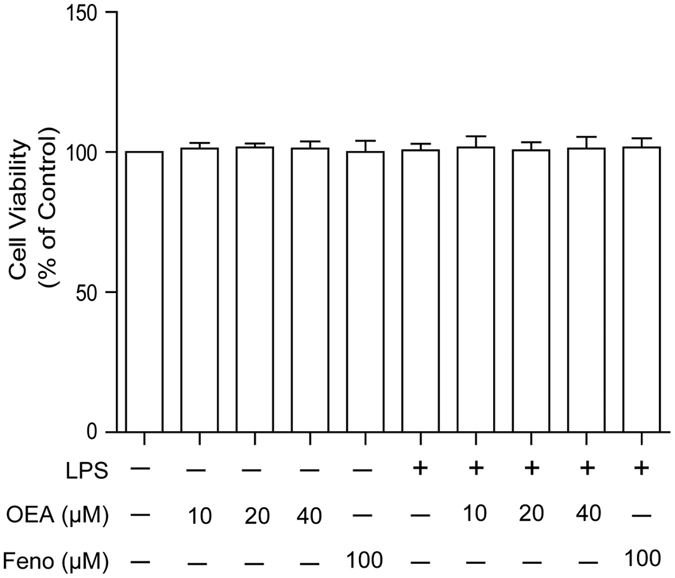
Effects of OEA and fenofibrate on human THP-1 cell viability. THP-1 cells were cultured with different concentrations of OEA (10, 20, and 40 μM) or fenofibrate (100 μM) in the absence or presence of 1 μg/ml LPS for 24 h. Cell viability was assessed using an MTT assay. The values are presented as means ± SEM of five independent experiments performed in duplicate (n = 5). ^#^*P* < 0.05 vs. control group.

The potential cytotoxicity of OEA was evaluated using the MTT assay. The cell viabilities were unaffected by OEA at all of the concentrations used (10, 20 and 40 μM) for 1 h before stimulation with or without LPS for another 24 h. Thus, the THP-1 cells treated with OEA (10, 20, 40 μM) or fenofibrate (100 μM) did not exhibit a reduction in viability excluding the potential effect of OEA cytotoxicity (Fig. 1).

### OEA attenuates TNF-α, IL-1β, and IL-6 production in LPS-stimulated THP-1 cells

**Figure 2:**
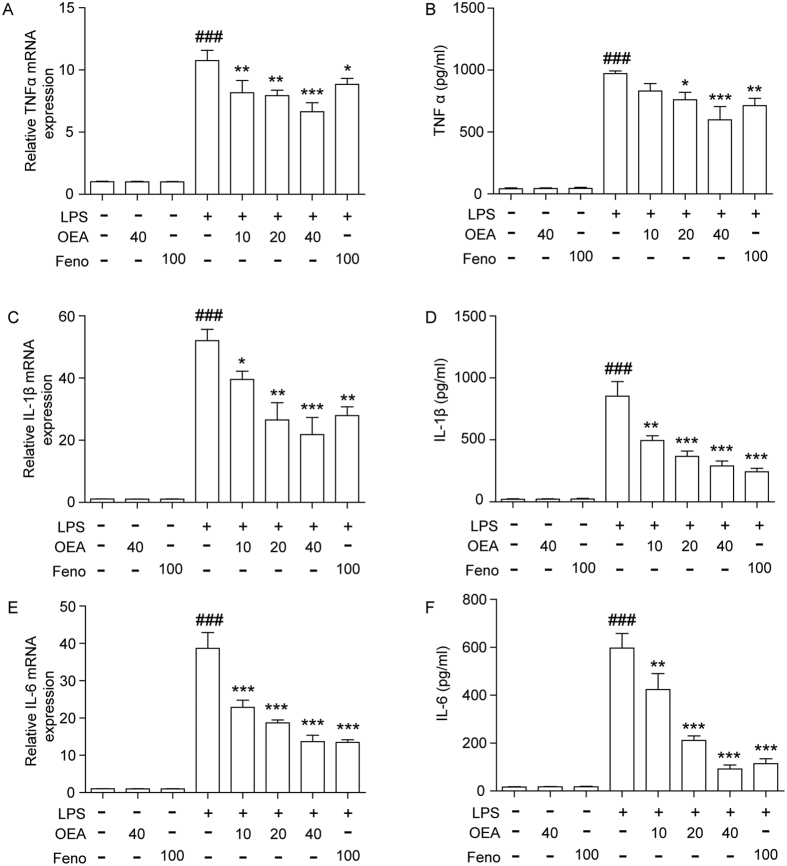
Inhibition of pro-inflammatory cytokine production through OEA and fenofibrate in LPS-induced THP-1 cells. The levels of cytokines TNF-α, IL-6, and IL-1β were measured using quantitative real-time PCR (**A,C,E**) or ELISA (**B,D,F**). The data are presented as means ± SEM of five independent experiments performed in duplicate (n = 5). ^###^*P* < 0.001 vs. control group, *P < 0.05, **P < 0.01, ***P < 0.001 vs. LPS-induced group.

The stimulation of THP-1 cells with LPS (1 μg/ml) markedly increased TNF-α, IL-1β, and IL-6 mRNA and protein expression. However, both pre-treatment or post-treatment of THP-1 cells with OEA and fenofibrate attenuated the LPS-stimulated pro-inflammatory cytokine mRNA expression (Fig. 2A,C,E and Supplemental Fig. 1). We did not observe that combined OEA and fenofibrate have a synergistic effect on anti-inflammation in LPS-induced THP-1 cells (Supplemental Fig. 2). The effects of OEA and fenofibrate on cytokine levels were measured by ELISA. The production of these pro-inflammatory mediators was significantly inhibited by OEA and fenofibrate pre-treatment (Fig. 2B,D,F). Furthermore, OEA or fenofibrate alone did not induce the production of TNF-α, IL-1β or IL-6. Additionally, the effect of OEA on inflammation *in vivo* was tested, and the results showed that the mRNA expression of TNFα, IL-1β and IL-6 in lung, liver, brain and spleen of LPS-treated mice was significantly increased compared with the vehicle-treated group. Furthermore, these inflammatory mediators were significantly inhibited by OEA and fenofibrate pre-treatment (Supplemental Fig. 3).

### OEA decreases the expression of TLR4 and increases the expression of PPARα in LPS-induced THP-1 cells

**Figure 3:**
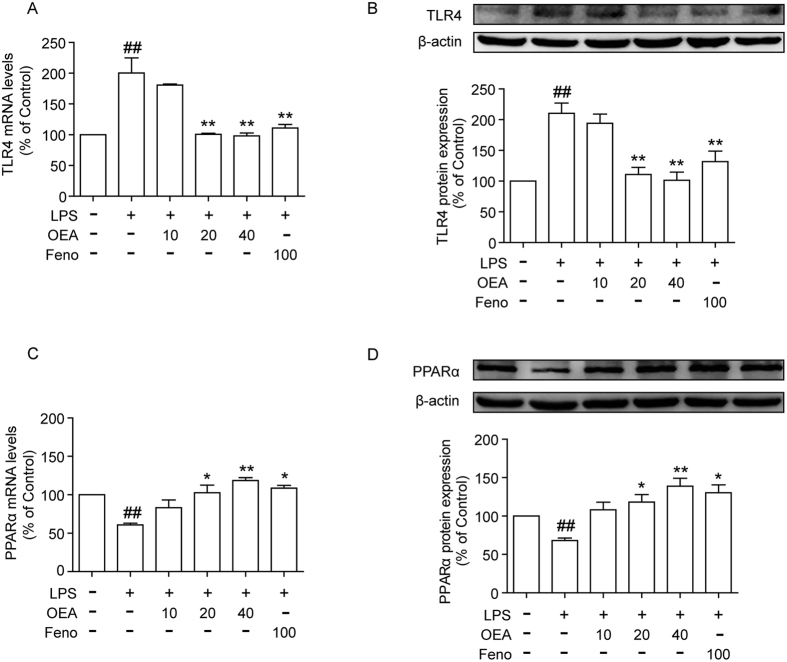
Effects of OEA and fenofibrate on LPS-induced TLR4 and PPARα mRNA and protein expression in THP-1 cells. The mRNA levels of TLR4 and PPARα were analyzed using quantitative real-time PCR after normalization to β-actin mRNA (**A,C**), and protein levels of TLR4 and PPARα were measured using western blot (**B,D**). The values are expressed as percentages compared with the control group (set to 100%) and represented as means ± SEM of five separate experiments performed in duplicate (n = 5). ^##^*P* < 0.01 vs. control group; *P < 0.05, **P < 0.01 vs. LPS-induced group.

We examined the effects of pre-treatment with OEA on LPS-induced TLR4 expression at both the mRNA and protein levels. The stimulation of THP-1 monocytes with LPS (1 μg/ml) significantly increased TLR4 expression compared with the controls, and pre-treatment with OEA and fenofibrate reduced the expression of TLR4 mRNA and protein (Fig. 3A,B). Because OEA is a natural agonist of PPARα, we further observed the effects of OEA on the expression of PPARα. When the THP-1 cells were stimulated with LPS alone, the PPARα mRNA levels were decreased compared with the controls, and different concentrations of OEA upregulated the expression of PPARα compared with the LPS-induced group. A similar effect was observed for fenofibrate (Fig. 3C). After 24 h simulation, the decreased levels of PPARα protein were similar to that of PPARα mRNA expression compared with the control group. However, OEA and fenofibrate pretreatment markedly increased PPARα protein expression (Fig. 3D). Meanwhile, we also tested the effect of OEA on PPARα expression in THP-1 cells. The results showed that the mRNA expression of PPARα in OEA pretreatment group was increased nearly 4 folds compared with the control group. This result suggested that OEA alone promote PPARα expression in THP-1 cells (as shown in Supplemental Fig. 5).

### OEA inhibition of inflammatory responses is dependent on PPARα

**Figure 4:**
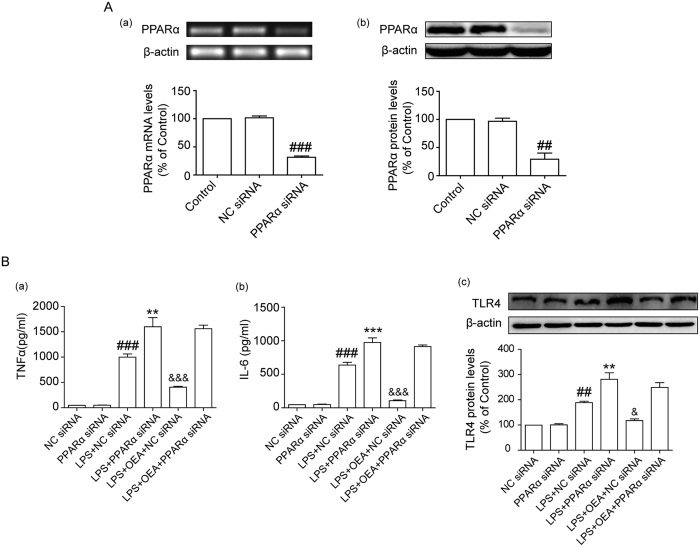
Effects of PPARα siRNA on the anti-inflammatory action of OEA in LPS-induced THP-1 cells. PPARα mRNA levels and protein expression in THP-1 cells were detected at 24 h post-transfection (**A**). The concentrations of TNF-α and IL-6 were determined using ELISA (**B**). TLR4 protein expression was detected through western blot (**B**). The results are presented as percentages compared with the control group or NC siRNA group (set to 100%) and represented as means ± SEM of five separate experiments performed in duplicate (n = 5). ^##^*P* < 0.01, ^###^*P* < 0.001 vs. NC siRNA; **P < 0.01, ***P < 0.001 vs. LPS + NC siRNA, ^&^*P* < 0.05, ^&&&^*P* < 0.001 vs. LPS + PPARα siRNA.

To elucidate the role of PPARα in the anti-inflammatory effects of OEA, THP-1 cells were transiently transfected with PPARα siRNA for 24 h, and the mRNA and protein expression levels of PPARα were subsequently measured (Fig. 4A). The knock-down efficiencies of PPARα were 69.7% and 71.3% as determined by quantitative real-time PCR and western blot analysis, respectively. Transfected THP-1 cells were pretreated with OEA (40 μM) for 1 h before exposure to LPS for another 24 h. As shown in Fig. 4B, stimulating the cells with LPS increased the TNF-α levels compared with the negative control, whereas reduced PPARα expression further promoted LPS-induced TNF-α production in THP-1 cells. These findings suggested that PPARα deficiency exacerbated LPS-induced TNF-α generation. OEA decreased LPS-induced TNF-α production in the negative control, but this effect was almost abrogated in the PPARα siRNA controls, which implies that inhibition of LPS-induced TNF-α production by OEA in THP-1 cells was dependent on the presence of PPARα. Results similar to those for TNF-α were observed for IL-6 (Fig. 4B). Furthermore, we found that PPARα siRNA exacerbated the LPS-elicited increase in TLR4 protein expression compared with the negative controls, indicating that PPARα signaling pathway might prevent the effects of LPS stimulation on TLR4 expression. OEA decreased TLR4 protein expression in the negative controls in LPS-stimulated THP-1 cells, but PPARα siRNA obviously antagonized the decreased effect of OEA on TLR4 protein expression (Fig. 4B). This finding indicated that the reduced TLR4 expression induced by OEA was PPARα dependent. These data demonstrated that OEA inhibits inflammatory responses in LPS-induced THP-1 cells via PPARα.

### OEA inhibition of LPS-mediated inflammatory responses in THP-1 cells is associated with TLR4

**Figure 5:**
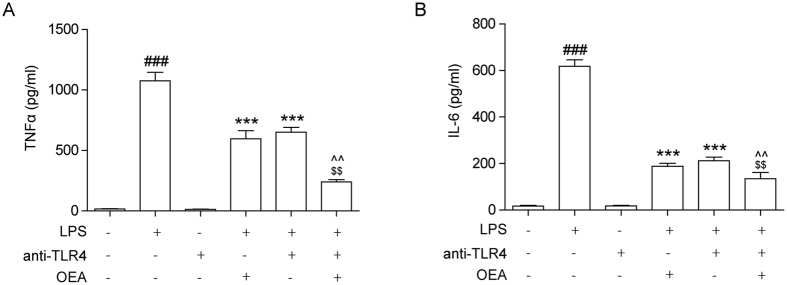
Association between the effects of OEA on LPS-induced inflammatory responses in THP-1 cells and TLR4. THP-1 cells were pretreated with anti-TLR4 antibody (3 μg/ml) for 0.5 h prior to the addition of OEA (40 μM) for 1 h, and subsequently stimulated with LPS (1 μg/ml) for 24 h. The conditioned media was collected, and TNF-α and IL-6 were measured using ELISA. The data are presented as means ± SEM from five independent experiments performed in duplicate (n = 5). ^###^*P* < 0.001 vs. control group; ***P < 0.001 vs. LPS-induced group; ^$$^*P* < 0.01 vs. LPS + OEA group; ^^^^*P* < 0.01 vs. LPS + anti-TLR4 group.

To examine whether OEA depresses LPS-induced inflammatory response via TLR4, THP-1 cells were pre-treated with anti-TLR4 monoclonal antibody (27 μM) for 1 h prior to the addition of OEA (40 μM) for 1 h and subsequently stimulated with LPS (1 μg/ml) for 24 h. The levels of TNF-α and IL-6 were detected by ELISA. The results revealed that LPS elicited TNF-α and IL-6 expression, whereas OEA and TLR4 inhibition remarkably reversed the LPS-induced effects on TNF-α and IL-6 in THP-1 cells. Furthermore, treatment of the cells with a combination of the TLR4 blocker and OEA synergistically reversed the effect elicited by LPS compared with the treatment with the anti-TLR4 antibody or OEA alone (Fig. 5). The TLR4 blocker neutralized the effect of LPS on TNF-α and IL-6 secretion, and OEA directly downregulated LPS-induced TLR4 expression; thus, the inhibitory effects of OEA on LPS-mediated inflammatory responses in THP-1 cells are dependent on TLR4.

### OEA attenuates the NF-κB inflammatory signaling pathway in LPS-induced THP-1 cells

**Figure 6:**
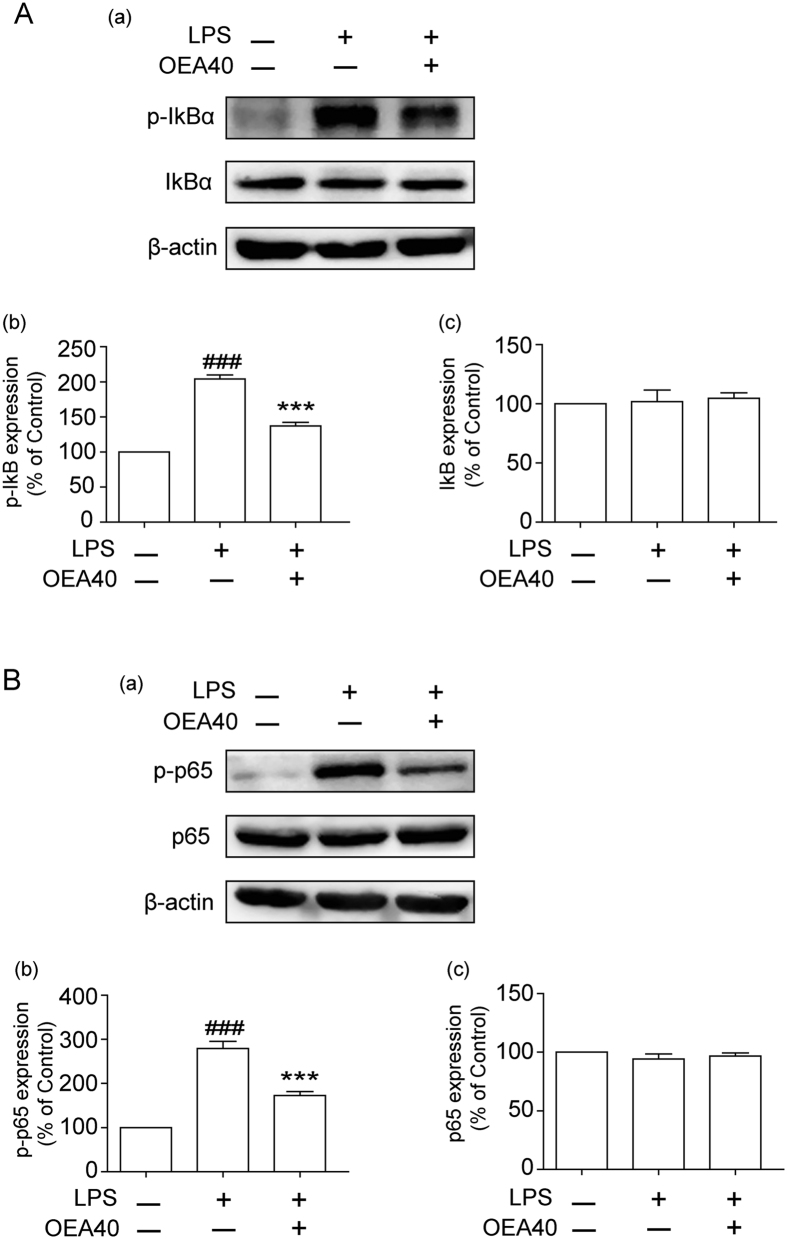
Effects of OEA on the NF-κB inflammatory signaling pathway in LPS-induced THP-1 cells. The cells were pretreated with OEA (40 μM) for 1 h and subsequently stimulated with LPS (1 μg/ml) for 24 h. The cell lysates were isolated through western blotting. The data are presented as percentages compared with the control group (set to 100%) and represented as means ± SEM of three or five separate experiments performed in duplicate (n = 3–5). ^###^*P* < 0.001 vs. control group; ***P < 0.001 vs. LPS-induced group.

The activation of NF-κB is a central event in the production of inflammatory cytokines. To evaluate whether the inhibition of inflammatory responses by OEA was mediated through the NF-κB pathway, inhibitory kappa Bα (IκBα) and NF-κB p65 proteins were measured by western blot. As illustrated in Fig. 6A, LPS markedly induced the phosphorylation of IκBα in THP-1 cells. However, these effects were significantly reversed by OEA (40 μM). We did not observe a significant difference in the IκBα levels among the groups. We further investigated the effect of OEA on NF-κB activation. Figure 6B demonstrates that the level of NF-κB p65 phosphorylation was significantly increased by LPS treatment of THP-1 cells. However, OEA reduced LPS-induced NF-κB p65 phosphorylation. Meanwhile, LPS markedly reduced IκB expression in LPS-stimulated THP-1 cells. However, these effects were significantly reversed by OEA (40 μM) at 60 min of LPS stimulation, which indicated that OEA inhibited LPS-induced IκBα degradation in THP-1 cells. In addition, a rapid increase in phosphorylation of IκBα and NF-κBp65 was observed at 15 min after LPS treatment. In contrast, OEA significantly blocked the changes in phosphorylation status of IκBα and p65 from 15 min and 30 min of LPS stimulation, respectively (Supplemental Figs 6A–C, and A–D). These findings indicate that OEA significantly inhibits the activation of NF-κB signaling pathway.

### OEA suppresses LPS-mediated inflammatory responses by inhibiting the ERK1/2/AP-1/STAT3 signaling pathway in THP-1 cells

**Figure 7:**
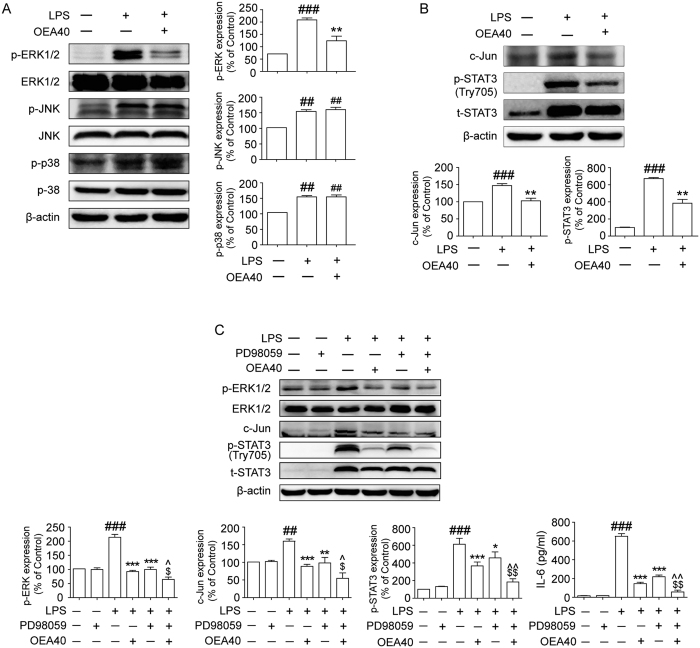
Effects of OEA on the ERK1/2/AP-1/STAT3 inflammatory signaling pathway in LPS-induced THP-1 cells. The cells were pretreated with OEA (40 μM) for 1 h and subsequently stimulated with LPS (1 μg/ml) for 24 h. The cell lysates were isolated through western blot. The data are presented as percentages compared with the control group (set to 100%) and represented as means ± SEM of five separate experiments performed in duplicate (n = 5). ^##^*P* < 0.01, ^###^*P* < 0.001 vs. control group; *P < 0.05, **P < 0.01, ***P < 0.001 vs. LPS-induced group; ^$^*P* < 0.05, ^$$^*P* < 0.01 vs. LPS + OEA group; ^^^*P* < 0.05, ^^^^*P* < 0.01 vs. LPS + PD group.

MAPKs also have pivotal roles in the induction of pro-inflammatory cytokine production. To investigate whether the inhibition of inflammatory responses by OEA is mediated through MAPK pathways, we measured the activation of ERK1/2, JNK and p38 MAPK by western blot. LPS markedly increased the phosphorylation of ERK1/2, JNK and p38 MAPK. Pre-treatment with OEA (40 μM) for 1 h significantly attenuated the activation of ERK1/2 that was induced by LPS. OEA did not have a significant effect on the phosphorylation of JNK or p38 MAPK, which suggests that these pathways are not involved in the inflammatory inhibiting effects of OEA. The total ERK1/2, JNK, and p38 MAPK levels were not different between the groups (Fig. 7A). However, OEA did inhibit the LPS-induced expression of the c-Jun subunit of AP-1 and the phosphorylation of STAT3 (Fig. 7B).

TLR4 signaling activates the pro-inflammatory transcription factor AP-1 through ERK-dependent phosphorylation^22^. To confirm the role of the ERK signal pathway in the anti-inflammatory mechanisms of OEA, THP-1 cells were subjected to PD098059 (1 μM) for 30 min followed by treatment with OEA (40 μM) for an additional 1 h and then stimulated with LPS (1 μg/ml) for 24 h. As evident in Fig. 7C, the ERK1/2 antagonist PD098059 inhibited the expression of the c-Jun subunit of AP-1, which suggests that the ERK signaling pathway is involved in AP-1 expression. Additionally, PD098059 pre-treatment indicated a role of the ERK1/2 pathway in the regulation of STAT3 protein induction by LPS. Thus, PD98059 attenuated the phosphorylation of STAT3 in response to LPS, which suggests that the ERK1/2 pathway plays a key role in the LPS-induced up-regulation of STAT3 activity in THP-1 cells. OEA also exerted similar effects on STAT3 phosphorylation levels and c-Jun expression with ERK1/2 blockade. Treatment of the cells with combined ERK1/2 blockade and OEA also synergistically reversed the LPS-mediated changes of STAT3 activation and c-Jun expression. Additionally, the ERK1/2 antagonist inhibited LPS-induced IL-6 production, and treatment with OEA alone had similar effects. Furthermore, treatment of the cells with combined ERK1/2 antagonist and OEA synergistically reversed the effects elicited by LPS compared with treatment using the ERK1/2 antagonist or OEA alone. Taken together, these findings reveal that the inhibitory effects of OEA on LPS-induced inflammatory responses in THP-1 cells are mediated through the ERK1/2/AP-1/STAT3 signaling pathway.

## Discussion

The major finding of this study is that the PPARα agonist OEA exerted a potent anti-inflammatory action by reducing the expression of inflammatory cytokines and TLR4 and by enhancing PPARα expression. We also illustrated that the modulatory effects of OEA on LPS-induced inflammation depended on both PPARα and TLR4. More importantly, our results demonstrate that OEA inhibits inflammatory responses by inhibiting the TLR4-mediated NF-κB signaling pathway and interfering with the ERK1/2-dependent signaling cascade (TLR4/ERK1/2/AP-1/STAT3).

The generation of pro-inflammatory cytokines by macrophages exposed to endotoxin is well established^23^. TNF-α has a central role in initiating and regulating the release of adhesion molecules and the expression of inflammatory mediators during inflammatory responses^24^. IL-1β is a major pro-inflammatory cytokine that mediates inflammatory responses at the local and systemic levels^25^. IL-6 is a multifunctional cytokine that has a major role in inflammatory responses^26^. These pro-inflammatory cytokines can cause severe septic shock and tissue damage. Therefore, treatments aimed at suppressing pro-inflammatory cytokines are important for the control of inflammatory diseases. Our results indicated that OEA efficiently suppressed TNF-α, IL-1β and IL-6 secretion and mRNA expression in LPS-stimulated human THP-1 cells. Interestingly, the inhibitory effects of OEA on IL-1β or IL-6 generation were greater than those of TNF-α, suggesting that OEA had a greater suppressive effect on the production of secondary cytokines. Furthermore, we tested the effect of OEA on inflammation *in vivo*, and the results showed that OEA pre-treatment significantly decreased the expression of TNFα, IL-1β and IL-6 in lung, liver, brain and spleen of LPS-treated mice (Supplemental Fig. 3). Therefore, OEA might be a promising candidate target to inhibit the inflammatory pathway.

TLR4 is an important member of the TLR family and is highly expressed on macrophages and recognizes LPS associated with gram-negative bacteria^27^. The activation of TLR4 by LPS elicits the release of major pro-inflammatory cytokines, and is a key etiological condition for the development of many chronic inflammatory diseases including atherosclerosis and diabetes^28^. Therefore, TLR4 signaling is receiving increased attention in the inflammation and atherosclerotic fields. In the present study, we observed that LPS upregulated TLR4 expression in THP-1 cells. However, treatment with OEA antagonized LPS-induced TLR4 expression, which provides direct evidence that PPARα agonists inhibit LPS-stimulated TLR4 expression. Other studies have shown that PPARα activators exert active effects on inflammation and immune responses^9^,^20^. Emerging evidence has shown that PPARα counter-regulate inflammatory responses. Our results revealed that OEA up-regulated PPARα expression in LPS-induced THP-1 cells. We further evaluated whether the anti-inflammatory activity of OEA was induced through PPARα. Consistent with previous studies^29^,^30^, our data indicated that a deficiency of the PPARα gene markedly accelerated the LPS-induced activation of inflammatory response genes. However, OEA did not exert anti-inflammatory effects in PPARα-silenced THP-1 cells, which suggests that OEA activity is directly dependent upon PPARα. Therefore, our results further confirmed that PPARα plays an essential role in the regulation of inflammatory responses. Additionally, a lack of PPARα exacerbated the LPS-elicited increases in the TLR4 protein, which demonstrates that PPARα signaling might interrupt the impact of LPS on TLR4. Taken together, these results indicate that the PPARα signaling pathway is a potentially interesting target for anti-inflammatory drug development. Although we demonstrated that OEA exerted a potent anti-inflammatory effect, the association between OEA effects on LPS-induced inflammatory responses and TLR4 is poorly understood. Therefore, TLR4 inhibition was applied to THP-1 cells to determine whether TLR4 was involved in the modulatory effect of OEA on LPS-induced inflammatory responses. Incubation with a TLR4-specific monoclonal antibody partially antagonized LPS-induced inflammatory responses in THP-1 cells that were potentiated by OEA. Therefore, our results indicate that the effects of LPS are mediated through TLR4, which agrees with the results of a previous study^3^. OEA also ameliorated the LPS-induced inflammatory responses and directly down-regulated LPS-mediated TLR4 expression in THP-1 cells, which further indicates that TLR4 is associated with the modulatory effect of OEA on LPS-induced inflammatory responses. Therefore, because the inhibition of TLR4 synergistically exacerbated the inhibitory effect of OEA on LPS-mediated inflammatory responses, TLR4 may be a new target for PPARα agonists in the regulation of LPS-induced inflammatory responses.

The activation of TLR4 signaling at the plasma membrane by LPS stimulated NF-κB signaling through the MyD88/TAK1 signaling complex. To evaluate the inhibitory mechanism of OEA on inflammatory responses, we measured IκBα phosphorylation, IκBα degradation, and NF-κB p65 activity. LPS stimulation markedly increased the phosphorylation and the activation of NF-κB p65 proteins. However, LPS-stimulated IκBα phosphorylation and NF-κB p65 activation were significantly reversed by pretreatment with OEA. Stimulation with LPS activated NF-κB via phosphorylation and regulated the expression of a network of inflammatory cytokines that included TNF-α, IL-1β and IL-6^2^. Indeed, PPARα activation effectively prevented the generation of inflammatory cytokines partially by negatively regulating NF-κB^9^,^31^. Because we showed that OEA enhanced PPARα expression in the current study, we speculate that OEA might inhibit NF-κB activity through PPARα. Thus, the OEA-mediated inhibition of NF-κB activation by LPS might be one possible mechanism underlying its inhibitory actions on pro-inflammatory cytokine production by macrophages.

LPS activates the TLR4-mediated signal pathway and leads to the activation of MAPKs to regulate the generation of pro-inflammatory mediators^32^. To further evaluate the inhibitory mechanism of inflammatory cytokine generation, the effects of OEA on MAPK activation were determined. We found that exposure to LPS induced a remarkable increase in the levels of phosphorylated ERK1/2, JNK, and p38 MAPK in THP-1 cells. In contrast, OEA treatment markedly reduced the activation of ERK1/2 induced by LPS, while the total protein levels of ERK1/2 remained unchanged, which indicates that OEA blocked the activation but not the biosynthesis of ERK1/2. Notably, OEA had no effect on the activation of JNK or p38 MAPK as induced by LPS, which suggests that these pathways are not involved in inflammatory response inhibition by OEA. The selectivity for OEA may be associated with cell specificity and the differential effects of MAPKs. Additionally, we observed that OEA reduced the LPS-mediated expression of AP-1. TLR4 signaling activates pro-inflammatory transcription factor AP-1 through ERK1/2-dependent phosphorylation^33^. In the present study, the ERK antagonist PD098059 inhibited the expression of AP-1, suggesting the ERK1/2 signaling pathway is involved in AP-1 in LPS-stimulated THP-1 cells. Previous studies have demonstrated that ERK1/2 phosphorylation activates STAT3^34^. Similarly, we demonstrated that the activation of TLR4 signaling in THP-1 cells upregulates STAT3 activation. Furthermore, this regulation was markedly attenuated by an ERK1/2 inhibitor, which demonstrated the phosphorylation of STAT3 through ERK1/2 activation. Moreover, treatment of the cells with combined ERK1/2 inhibitor and OEA synergistically reversed LPS-mediated changes of STAT3 activation and AP-1 expression, which suggests that OEA inhibits STAT3 activity and AP-1 expression via ERK1/2 signaling. Previous reports demonstrated that the generation of IL-1β and IL-6 are predominantly affected by ERK1/2, whereas TNF-α is regulated by JNK after LPS stimulation^35^,^36^. STAT3 is important for the production of pro-inflammatory cytokines, such as IL-1β and IL-6^37^. In the present study, IL-6 was chosen as a marker to evaluate the ERK1/2/STAT3 signaling pathway after OEA inhibition of the inflammatory response. We found that ERK1/2 blockade reduced LPS-induced IL-6 generation. Furthermore, treatment of the cells with combined ERK1/2 inhibition and OEA also synergistically reversed LPS-mediated IL-6 production. Therefore, activation of the STAT3 pathway is associated with IL-6 induction by ERK1/2 activation in THP-1 cells, and this might contribute to an improved understanding of the overall regulating process of IL-6 expression through TLR4 signaling. Therefore, OEA exerted anti-inflammatory effects that were mediated at least in part, by interfering with TLR4, and by modulating the ERK1/2/AP-1/STAT3 signaling pathway.

**Figure 8:**
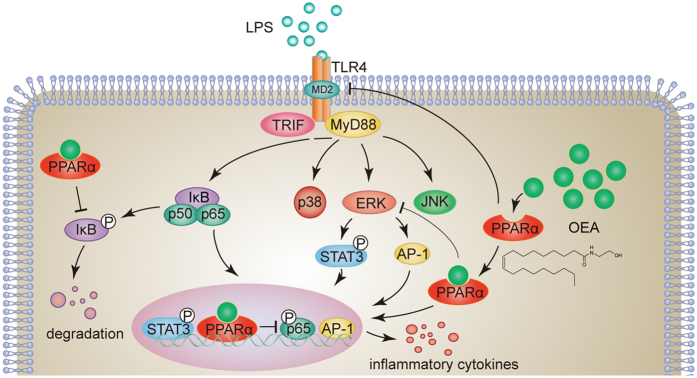
Mechanism of OEA inhibition of inflammation in LPS-treated THP-1 cells. OEA suppressed pro-inflammatory cytokine production through PPARα and the TLR4, suggesting a potential mechanism for the inhibition of NF-κB and ERK1/2/AP-1/STAT3 signaling pathways.

In conclusion, the present study provides strong evidence that OEA negatively controls inflammatory responses and prevents the repression of PPARα expression triggered by LPS. Our results suggest that OEA suppresses pro-inflammatory cytokine generation by enhancing PPARα signaling and downregulating TLR4 expression, which might inhibit the NF-κB and ERK1/2/AP-1/STAT3 signaling pathways (Fig. 8). These findings provide further support for current clinical trials aimed at assessing the beneficial effects of OEA administration against chronic inflammatory diseases.

## Methods

### Reagents

Dulbecco’s modified Eagle’s medium (DMEM), foetal bovine serum (FBS), penicillin and streptomycin were purchased from Gibco BRL (Carlsbad, CA, USA). LPS (L4391), OEA, fenofibrate, and PD98059 were purchased from Sigma (St. Louis, MO, USA). Monoclonal anti-TLR4 antibody (MTS510) was purchased from eBioscience (San Diego, CA, USA). Antibodies against TLR4 (1:500, Cell Signaling Technology, Boston, USA), PPARα (1:1000, Abcam, Cambridge, UK), phospho-IκBα (Ser32) (1:1000, Cell Signaling Technology), IκBα (1:1000, Cell Signaling Technology), phospho-NF-κB p65 (1:1000, Cell Signaling Technology), NF-κB p65 (1:1000, Cell Signaling Technology), AP-1 (c-Jun) (1:1000, Cell Signaling Technology), total-p38 (1:1000, Cell Signaling Technology), phospho-p38(T180 + Y182) (1:1000, Cell Signaling Technology), total-ERK (1:1000, Cell Signaling Technology), phospho-ERK (Thr202/Thr204) (1:1000, Cell Signaling Technology), total-JNK (1:1000, Cell Signaling Technology), phospho-JNK (Thr183 + Thr185) (1:1000, Cell Signaling Technology), total-STAT3 (1:1000, Cell Signaling Technology), phospho-STAT3 (Tyr705) (1:1000, Cell Signaling Technology), and mouse monoclonal anti-β-actin (1:10000, Sigma) were obtained from the manufacturers listed. TNF-α, IL-1β, and IL-6 ELISA kits were purchased from Bender (Bender MedSystems, San Diego, CA, USA), siRNA specific for PPARα (stealth RNAi select) and negative control siRNA (12935-200) and Lipofectamine™ RNAiMAX Transfection Reagent (13778-075) were purchased from Invitrogen Life Technologies (Carlsbad, CA, USA).

### Cell culture

THP-1 cells (donated by the Fujian Medical University) were cultured in RPIM-1640 supplemented with 10% FBS, 5 × 10^−5 ^mol/L 2-mercaptoethanol, 100 U/ml penicillin, and 100 μg/ml streptomycin and subcultured in suspension every 2 days until passage. In all experiments, the THP-1 cells were incubated in the presence or absence of OEA and fenofibrate, which were added 1 h prior to LPS (1 μg/ml) treatment. OEA and fenofibrate were dissolved in dimethyl sulfoxide (DMSO), and the final DMSO concentration was less than 0.05%.

### Animals and treatments

All experimental procedures were performed in accordance with the guidelines for animal care and use and were approved by the Committee for Animal Research at Xiamen University, and all efforts were made to minimize pain and suffering in the animals. Male ICR mice weighing 20–22 g were purchased from Beijing Vitalriver Experimental Animal Co. (Beijing, China) and housed under a 12/12 h dark/light cycle and specific pathogen-free (SPF) conditions. The animals were fasted without food deprivation for 12 h before the experiment was performed. Mice were randomly divided into varying groups: (i) Control group received 0.9% physiological saline; (ii) LPS model group (20 mg/kg, i.p.); (iii) Drug treatment group (OEA10, 20, 40 mg/kg and fenofibrate 100 mg/kg, i.p). Mice were pre-treated with different concentrations of OEA and fenofibrate for 1 h prior to stimulation with LPS for another 3 h, then the animals were sacrificed. Each group consisted of eight animals. After sacrifice, gene expression of TNFα, IL-1β and IL-6 was assessed in lung, liver, brain and spleen using real-time quantitative PCR.

### RT-PCR and quantitative real-time PCR

The THP-1 cells were pretreated with varying concentrations of OEA (10, 20, and 40 μM) and fenofibrate (100 μM) or 0.04% DMSO for 1 h before exposure to LPS for an additional 6 h or the THP-1 cells were stimulated with LPS for 1 h then post-treated with OEA and fenofibrate for another 5 h. Total RNA was extracted using TRIzol reagent, and reverse transcriptions were performed in a 20-μl mixture with 1 μg of total RNA according to the manufacturer’s instructions. The oligonucleotide primers used for PCR amplification that were obtained from target cellular RNA are listed in Supplemental Fig. 4. PCR amplification consisted of 30 cycles of 2 min of denaturation at 95 °C, 45 s of annealing at 60 °C, and 2 min of extension at 72 °C.

Real-time PCR was conducted with Fast Start Universal SYBR Green Master (Roche Applied Science, Penzberg, Germany) on an ABI PRISM 7300 Sequence Detection System. The TNF-α, IL-6, IL-1β, PPAR-α and TLR4 gene expression C_t_ values were normalized to the corresponding values for β-actin gene expression. Primer sequences for quantitative real-time PCR assay are showed in Supplemental Fig. 4.

### Enzyme-linked immunosorbent assay (ELISA)

To determine the expression of pro-inflammation cytokines, 1 × 10^6^ THP-1 cells/ml were placed in 12-well plates, pretreated with different concentrations of OEA and fenofibrate for 1 h prior to stimulation with LPS for another 24 h. The levels of TNF-α, IL-6, IL-1β in the culture supernatants were measured using commercial ELISA kits according to the manufacturer’s instructions (Bender Med Systems). In another experiment, the cells were pretreated with anti-TLR4 antibody (3 μg/ml) for 0.5 h prior to the addition of OEA for 1 h and subsequently stimulated with LPS (1 μg/ml) for 24 h.

### Western blot analysis

The protein samples (30 μg) were separated on 10% SDS-PAGE gels and transferred onto polyvinylidene difluoride membranes (Millipore, Billerica, MA, USA). The membranes were blocked with 5% BSA in Tris-buffered saline containing 0.1% Tween 20 and incubated with specific antibodies. The values were normalized to the β-actin intensity levels. Immunoreactive proteins were detected with an enhanced chemiluminescence (ECL) kit (Millipore, Billeria), the relative density of the protein bands was scanned using an Image Station 4000R (Rochester, New York, USA), and analyzed by densitometric evaluation using the Quantity-One software (Bio-Rad Hercules, CA, USA).

### siRNA transfection

A siRNA strategy was employed to silence PPARα in THP-1 cells. Overall, 1 × 10^5^ cells were diluted in fresh medium without antibiotics and transferred to 24-well plates 24 h before transfection. For transfection, all siRNAs (PPARα or the negative control) were re-suspended to a final concentration of 20 nM. After 24 h incubation, the transfected cells were pre-treated with 40 μM OEA for 1 h before stimulation with 1 μg/ml LPS. The total proteins were isolated, and the supernatants were collected after 24 h stimulation. A transfection rate of 65–75% of cells was used for all of the experiments.

### Statistical analysis

Results are expressed as means ± standard error (SEM). Data were analysed by one-way analysis of variance (ANOVA) of the differences within treatments followed by Tukey’s *post hoc* test (Prism 5 for Windows, GraphPad Software Inc., USA). *P* < 0.05 was considered to be significant.

## Additional Information

**How to cite this article**: Yang, L. *et al.* Oleoylethanolamide exerts anti-inflammatory effects on LPS-induced THP-1 cells by enhancing PPARα signaling and inhibiting the NF-κB and ERK1/2/AP-1/STAT3 pathways. *Sci. Rep.* **6**, 34611; doi: 10.1038/srep34611 (2016).

## Supplementary Material

Supplementary Information
