# Supplementary material for: Oleoylethanolamide exerts anti-inflammatory effects on LPS-induced THP-1 cells by enhancing PPARα signaling and inhibiting the NF-κB and ERK1/2/AP-1/STAT3 pathways
Source: Sci Rep. 2016 Oct 10;6:34611. doi: 10.1038/srep34611 (PMC5056375; doi:10.1038/srep34611)
Supplement: Supplementary Information [file srep34611-s1.doc]

**Oleoylethanolamide exerts anti-inflammatory effects on LPS-induced THP-1 cells by enhancing PPARα signaling and inhibiting the NF-κB and ERK1/2/AP-1/STAT3 pathways**

Lichao Yang^1,+^, Han Guo^1,+^, Ying Li^2,+^, Xianglan Meng^1^, Lu Yan^1^, Dan Zhang^3^, Sangang Wu^4^, Hao Zhou^1^, Lu Peng^1^, Qiang Xie^3,*^ & Xin Jin^1,*^

^1^Xiamen Key Laboratory of Chiral Drugs, Medical College, Xiamen University, Xiamen, Fujian, 361102, P. R. China. ^2^Department of Pharmacology, Xiamen Medical College, Xiamen, Fujian, 361008, P. R. China. ^3^Department of Cardiology, the First Affiliated Hospital of Xiamen University, Xiamen, Fujian, 361003, P. R. China. ^4^Xiamen Cancer Center, Department of Radiation Oncology, the First Affiliated Hospital of Xiamen University, Xiamen, Fujian, 361003, P. R. China. ^+^These authors contributed equally to this work and should be considered co-first authors. Correspondence and requests for materials should be addressed to Q.X. (email: [arther2014@sina.com](mailto:arther2014@sina.com)) or X.J. (email: xinjin@xmu.edu.cn)


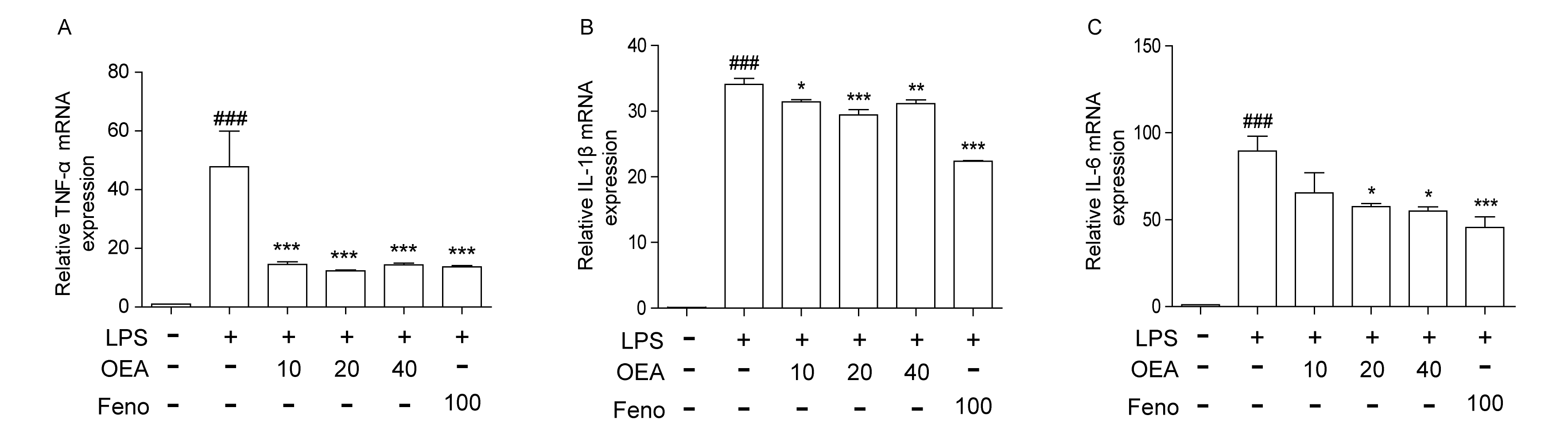


Supplemental figure 1. OEA post-treatment inhibits LPS-induced cytokines production. The THP-1 cells stimulated with LPS for 1 h then post-treatment with OEA and fenofibrate for another 5 h. The expression of TNF-α, IL-6, and IL-1β were measured using quantitative real-time PCR (A, B, and C). The data are presented as means ± SEM of five independent experiments performed in duplicate (n=5). ^###^*P*<0.001 vs. control group, ^*^*P*<0.05, ^**^*P*<0.01, ^***^*P*<0.001 vs. LPS-induced group.


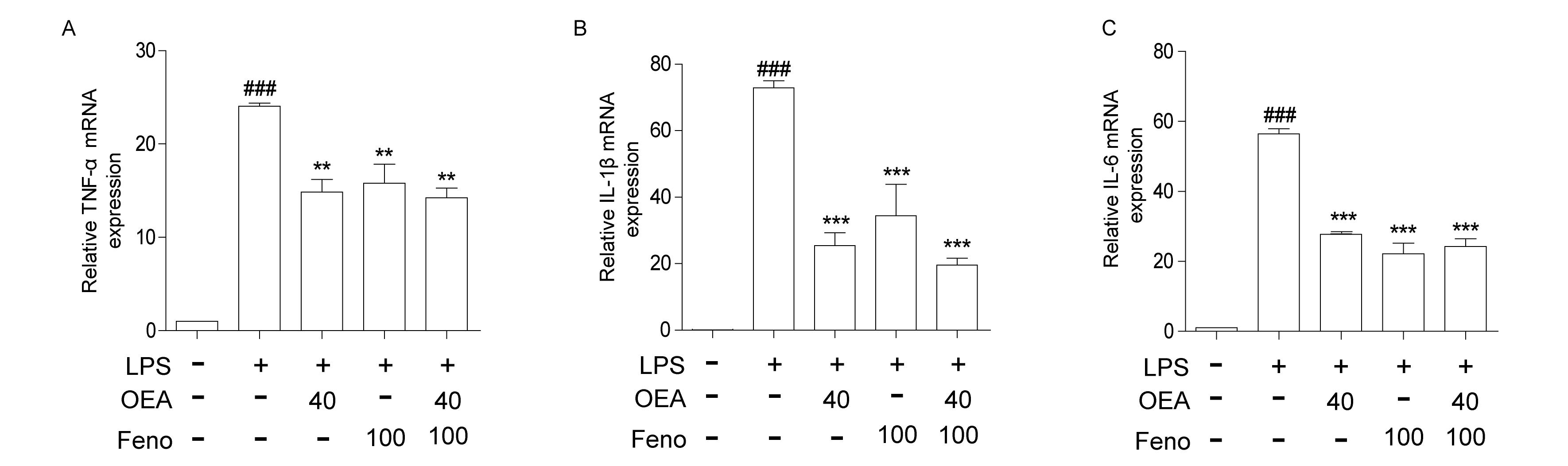


Supplemental figure 2. Effects of combined OEA and fenofibrate on LPS-induced inflammatory cytokines production. The levels of cytokines TNF-α, IL-6, and IL-1β were measured using quantitative real-time PCR (A, B, and C). The data are presented as means ± SEM of five independent experiments performed in duplicate (n=5). ^###^*P*<0.001 vs. control group, ^**^*P*<0.01, ^***^*P*<0.001 vs. LPS-induced group.


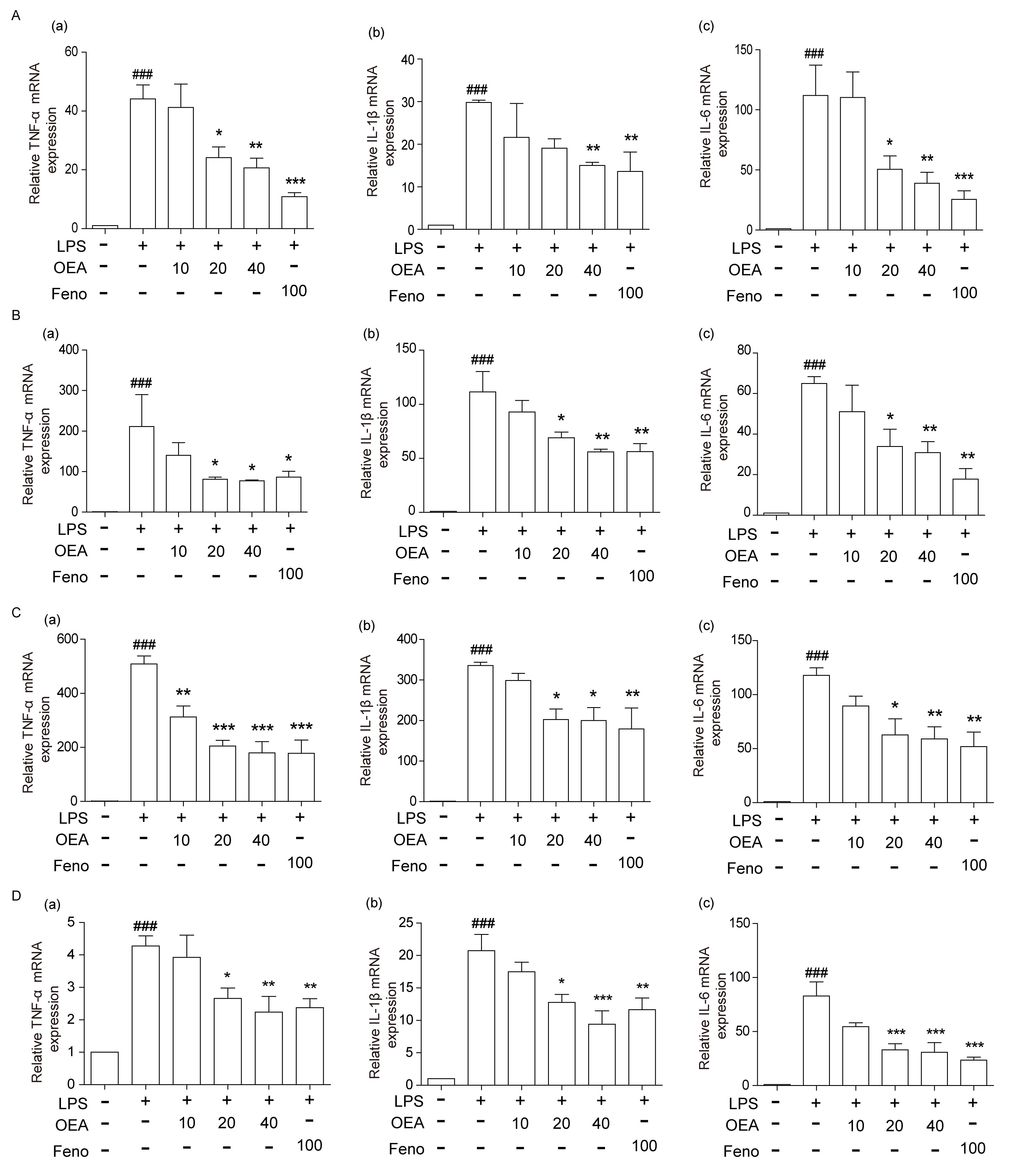


Supplemental figure 3. Inhibition of pro-inflammatory cytokine production through OEA and fenofibrate in LPS-induced mice. Mice pretreated with different concentrations of OEA and fenofibrate for 1 h prior to stimulation with LPS for another 3 h. Quantitative real-time PCR analysis was performed for TNF-α, IL-6, and IL-1β mRNA in in lung, liver, brain and spleen. The data are presented as means ± SEM of five independent experiments performed in duplicate (n=8). ^###^*P*<0.001 vs. control group, ^*^*P*<0.05, ^**^*P*<0.01, ^***^*P*<0.001 vs. LPS-induced group.


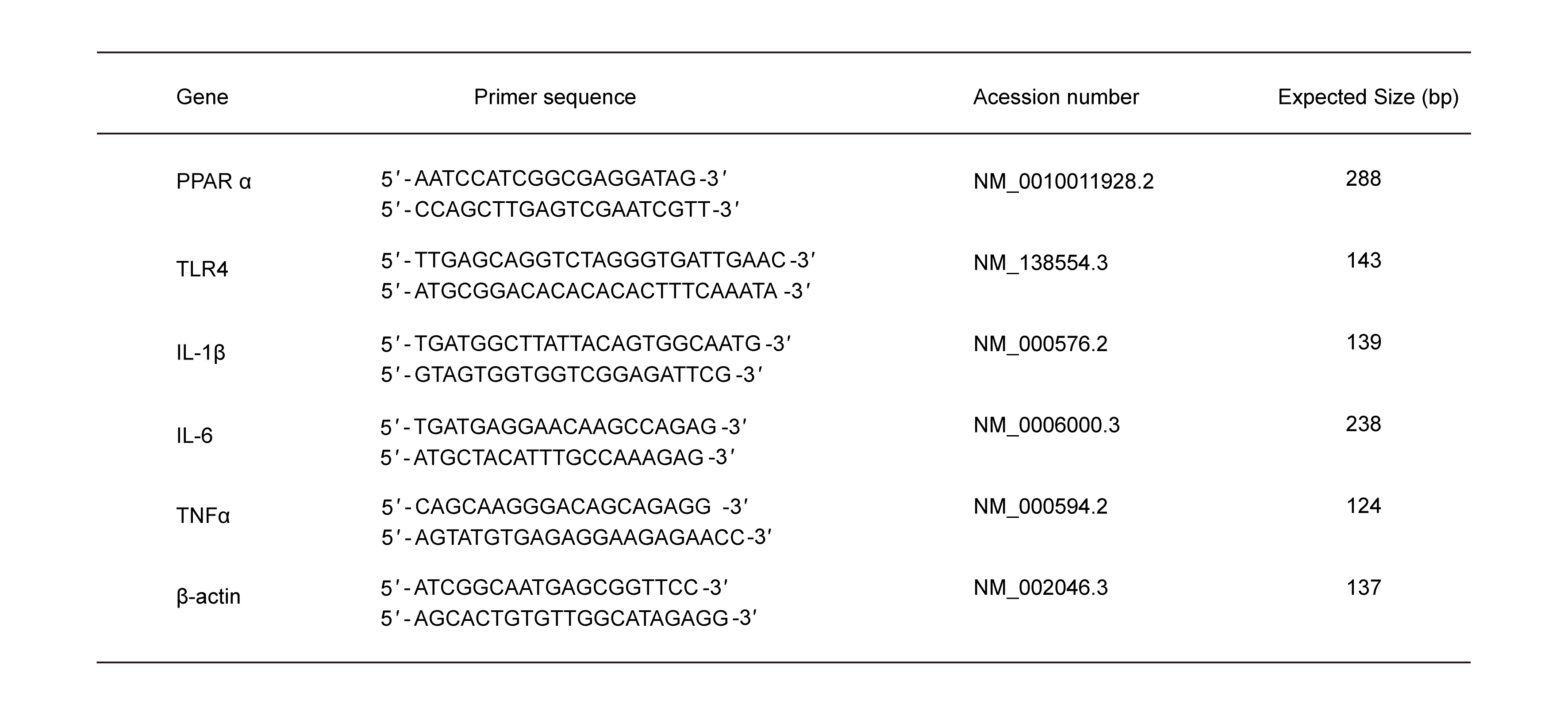


Supplemental figure 4. Primer sequences for quantitative real-time PCR assay.


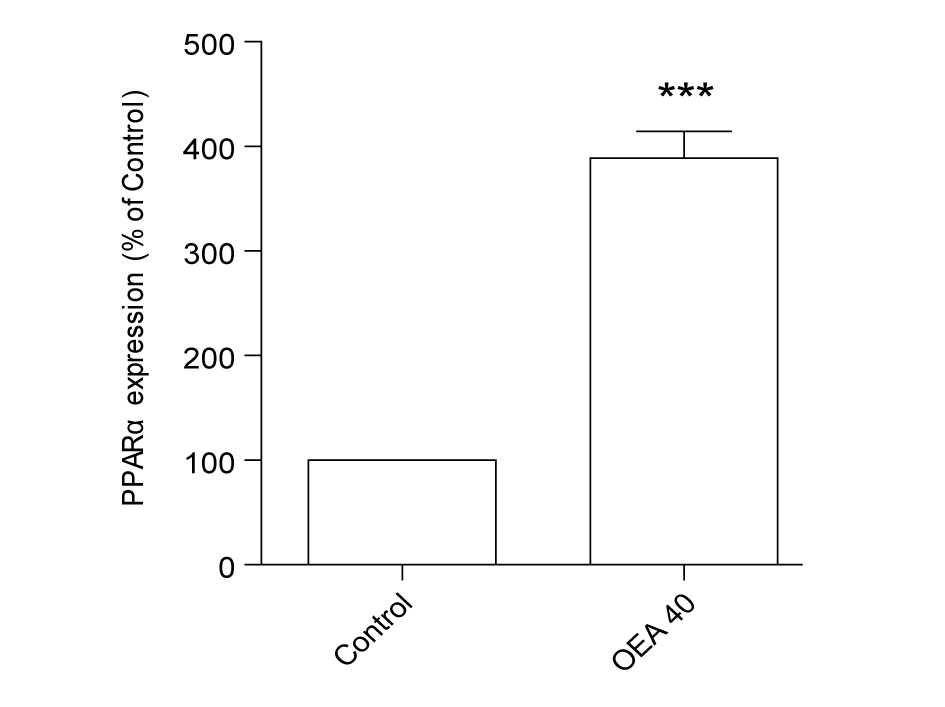


Supplemental figure 5. Effect of OEA on PPARα expression in THP-1 cells. The levels of PPARα was tested using quantitative real-time PCR. The results are presented as percentages compared with the control group (set to 100%) and represented as means ± SEM of three separate experiments (n=3). ^###^*P*<0.001 vs. control group.


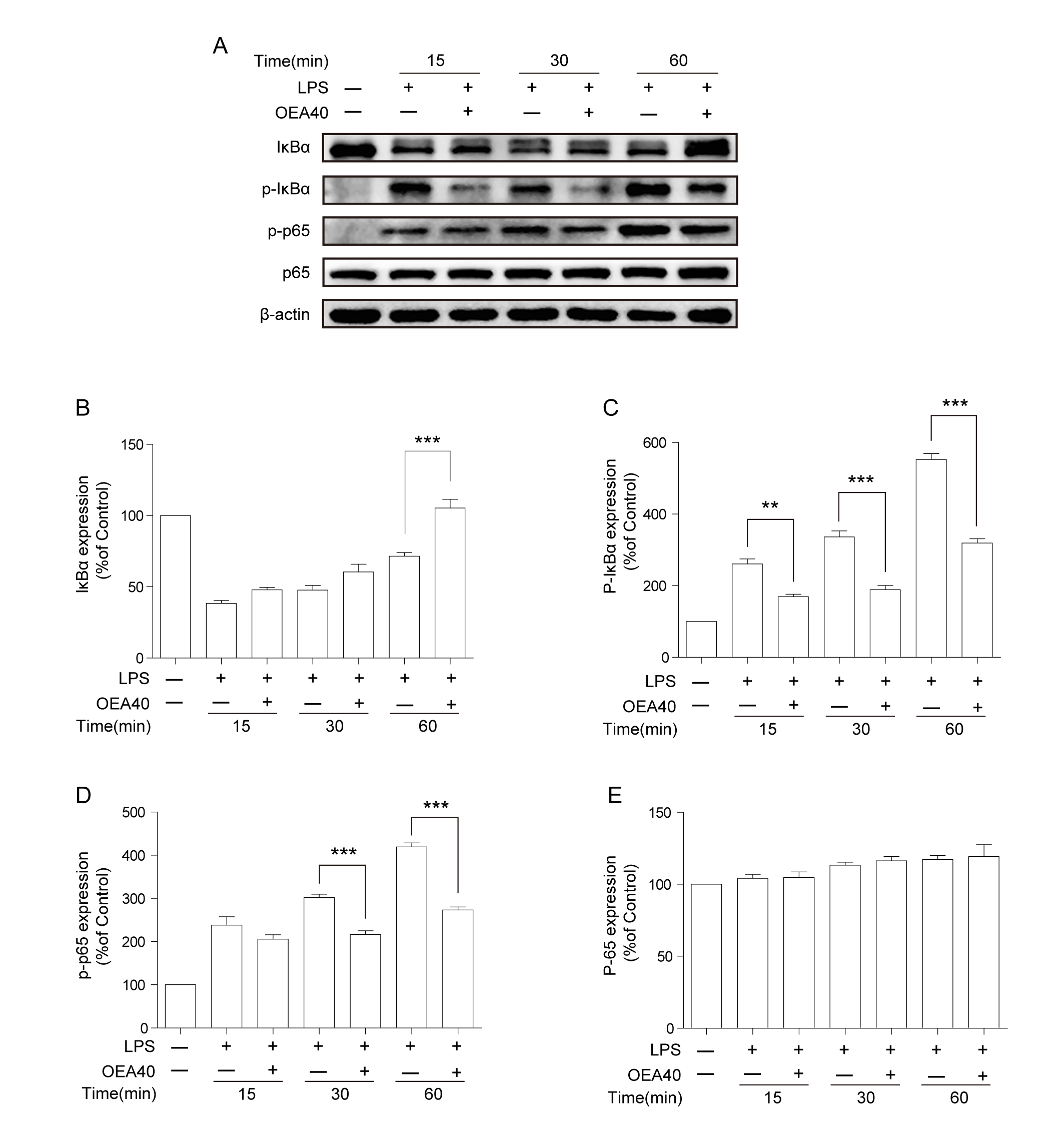


Supplemental figure 6. OEA bloked IκBα and NF-κBp65 activation in LPS-stimulated THP-1 cells. Cells were treated with OEA (40 μM) in absence or presence of LPS (1μg/ml) for the indicated time. The protein level of were detected by Western blotting analyses, β-actin was used as internal loading control. The data are presented as percentages compared with the control group (set to 100%) and represented as means ± SEM of three separate experiments performed in duplicate (n=3). Representative results of immunoblots (A) and their quantifications (B, C, D, and E) to better view the difference between different treatment groups were shown. The data are presented as means ± SEM. ^**^*P*<0.01, ^***^*P*<0.001 vs. LPS-induced group.

**Figure 3 full-length gel and blots**


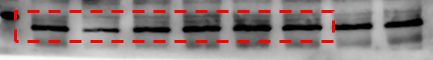
PPARα (53 kDa)


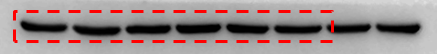
β-actin (44 kDa)


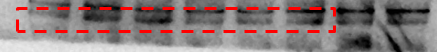
TLR4(95 kDa)


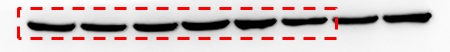
β-actin (44 kDa)

**Figure 4 full-length gel and blots**


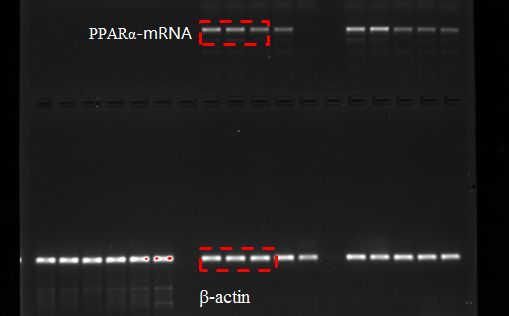


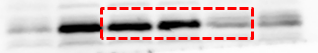
PPARα (53 kDa)


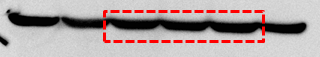
β-actin (44 kDa)


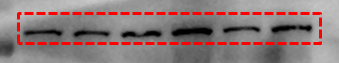
TLR4(95 kDa)


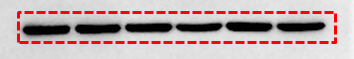
β-actin (44 kDa)

**Figure 6 full-length gel and blots**


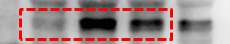
p-IκBα(39 kDa)


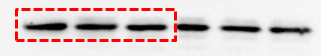
IκBα(39 kDa)


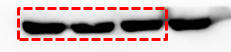
β-actin (44 kDa)


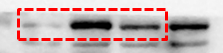
p-p65(65 kDa)


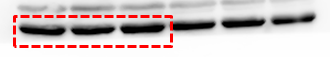
p65(65 kDa)


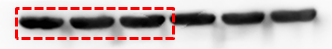
β-actin (44 kDa)

**Figure 7 full-length gel and blots**

**7A-a**


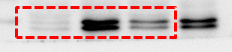
p-ERK1/2(42,44 kDa)


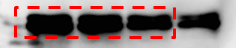
ERK1/2(42,44 kDa)


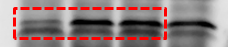
p-JNK(46 kDa)


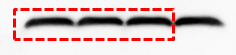
JNK(46 kDa)


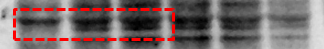
p-p38(43 kDa)


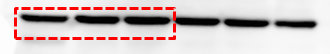
p38(43 kDa)


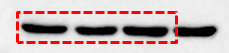
β-actin (44 kDa)

**7A-b**


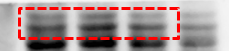
c-Jun(48 kDa)


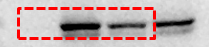
p-STAT3(88 kDa)


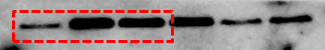
 STAT3(88 kDa)


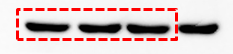
β-actin (44 kDa)

**7A-c**


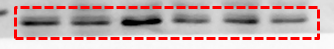
p-ERK1/2(42,44 kDa)


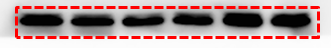
ERK1/2(42,44 kDa)


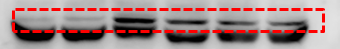
 c-Jun(48 kDa)


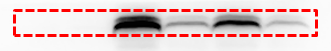
p-STAT3(88 kDa)


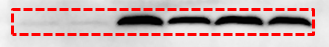
STAT3(88 kDa)


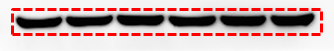
β-actin (44 kDa)

**Supplementary Figure. 6 full-length gel and blots**


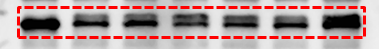
IκBα(39 kDa)


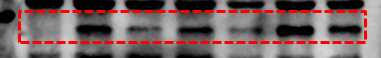
p-IκBα(39 kDa)


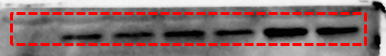
p-p65(65 kDa)


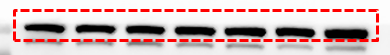
p65(65 kDa)


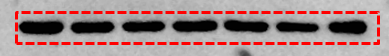
β-actin (44 kDa)
